# Retraction: Long non-coding RNA SNHG12, a new therapeutic target, regulates miR-199a-5p/Klotho to promote the growth and metastasis of intrahepatic cholangiocarcinoma cells

**DOI:** 10.3389/fmed.2026.2038820

**Published:** 2026-10-30

**Authors:** Frontiers Editorial Office

The journal retracts the 22 June 2021 article cited above.

Following publication, concerns were raised regarding the integrity of the images in the published figures. The authors failed to provide the raw data or a satisfactory explanation during the investigation, which was conducted in accordance with Frontiers' policies. Given the concerns about the validity of the data, and the lack of raw data, the editors no longer have confidence in the findings presented in the article.

This retraction was approved by the Chief Editors of Frontiers in Medicine and the Chief Executive Editor of Frontiers. The authors received a communication regarding the retraction and had a chance to respond. This communication has been recorded by the publisher.

